# Somatic Copy Number Alteration in Circulating Tumor DNA for Monitoring of Pediatric Patients with Cancer

**DOI:** 10.3390/biomedicines11041082

**Published:** 2023-04-03

**Authors:** Juliana Silveira Ruas, Felipe Luz Torres Silva, Mayara Ferreira Euzébio, Tássia Oliveira Biazon, Camila Maia Martin Daiggi, Daniel Nava, Mayra Troiani Franco, Izilda Aparecida Cardinalli, Alejandro Enzo Cassone, Luiz Henrique Pereira, Ana Luiza Seidinger, Mariana Maschietto, Patricia Yoshioka Jotta

**Affiliations:** 1 Research Center, Boldrini Children’s Hospital, Campinas 13083-884, SP, Brazil; ruasjulianas@gmail.com (J.S.R.); felipeluztorres@outlook.com (F.L.T.S.); ma.euzebio@gmail.com (M.F.E.); tassiabiazon@gmail.com (T.O.B.); analuseidinger@gmail.com (A.L.S.); marianamasc@gmail.com (M.M.); 2 Genetics and Molecular Biology, Institute of Biology, State University of Campinas, Campinas 13083-862, SP, Brazil; 3 Boldrini Children’s Hospital, Campinas 13083-210, SP, Brazil; camilammdaiggi@gmail.com (C.M.M.D.); danielnava@gmail.com (D.N.); mayra.franco@boldrini.org.br (M.T.F.); izilda.cardinalli@boldrini.org.br (I.A.C.); alejandro.cassone@boldrini.org.br (A.E.C.); drluizhpereira@gmail.com (L.H.P.)

**Keywords:** cell-free DNA, circulating tumor DNA, copy number alterations, digital PCR, *MYCN*, 1q

## Abstract

Pediatric tumors share few recurrent mutations and are instead characterized by copy number alterations (CNAs). The cell-free DNA (cfDNA) is a prominent source for the detection of cancer-specific biomarkers in plasma. We profiled CNAs in the tumor tissues for further evaluation of alterations in 1q, *MYCN* and 17p in the circulating tumor DNA (ctDNA) in the peripheral blood at diagnosis and follow-up using digital PCR. We report that among the different kinds of tumors (neuroblastoma, Wilms tumor, Ewing sarcoma, rhabdomyosarcoma, leiomyosarcoma, osteosarcoma and benign teratoma), neuroblastoma presented the greatest amount of cfDNA, in correlation with tumor volume. Considering all tumors, cfDNA levels correlated with tumor stage, metastasis at diagnosis and metastasis developed during therapy. In the tumor tissue, at least one CNA (at *CRABP2*, *TP53*, surrogate markers for 1q and 17p, respectively, and *MYCN*) was observed in 89% of patients. At diagnosis, CNAs levels were concordant between tumor and ctDNA in 56% of the cases, and for the remaining 44%, 91.4% of the CNAs were present only in cfDNA and 8.6% only in the tumor. Within the cfDNA, we observed that 46% and 23% of the patients had *MYCN* and 1q gain, respectively. The use of specific CNAs as targets for liquid biopsy in pediatric patients with cancer can improve diagnosis and should be considered for monitoring of the disease response.

## 1. Introduction

Pediatric tumors represent a heterogeneous group of malignancies, including embryonic tumors, which are composed of undifferentiated cells left over from the early stages of embryo development [1]. These tumors can arise in different organs and typically affect children and young adults. The most common types of embryonal tumors are neuroblastoma, Wilms tumor, retinoblastoma, hepatoblastoma, central nervous system tumors and several kinds of sarcoma [2,3,4]. Given their lack of differentiation as well as their elevated growth potential, this group of tumors may behave aggressively, and treatment approaches have had variable success, depending on tumor type [4]. Molecular profiles obtained from these pediatric tumors are typically characterized by a relatively low mutational burden, with a high prevalence of structural variations, e.g., chromosomal rearrangements and copy number alterations (CNAs) driving tumorigenesis [5,6,7]. Solid pediatric cancers besides embryonic tumors include some sarcomas (including osteosarcomas), central nervous system tumors and, more rarely, histologies usually found in adults [8].

CNAs have been profiled in thousands of tumor samples in virtually all cancer types [9]. In a series of 1699 pediatric tumors, 62% of the alterations were located in a CNA with many of them containing genes associated with cancer [8]. For several cancers, the chromosomal copy number profile of tumor tissue is not only prognostic but also a predictive biomarker of therapeutic response. For Wilms tumors, 1q and *MYCN* gains, as well as 17p loss, were correlated with a poor outcome [10,11,12,13]. For neuroblastoma, *MYCN* amplification, 1q gain as well as 1p and 11q deletions were independent predictors of decreased overall survival [14]. Although rare, CNAs have been reported in sarcomas and may be associated with a poor prognosis [15,16]. In particular, *MYCN* gain was associated with an adverse prognosis in patients with rhabdomyosarcoma [17].

Recurrent CNAs in *MYCN*, 1q and 17p loci are considered good biomarkers for cancer monitoring through circulating tumor DNA (ctDNA) detection in liquid biopsy since they are reported in several pediatric malignancies [18,19,20,21,22,23,24] and can be identified using accessible methodologies, such as digital PCR (dPCR) and qPCR [25]. Although dPCR provides limited information regarding the profile of alterations, it has a low cost and high sensitivity. Therefore, once the target regions are identified, great precision can readily be achieved with the depth of dPCR [26]. Although CNAs are present in several cancers, their measurement or even identification can be attenuated by sample heterogeneity, meaning precise measurements of this kind are required to discriminate small differences from normal [27].

ctDNA levels are associated with the prognosis and may assist in the disease monitoring of patients diagnosed with a wide range of malignancies, e.g., prostate, breast, bladder and ovarian cancer [25,28,29,30,31,32,33]. In patients diagnosed with osteosarcoma, higher ctDNA levels were associated with a worse prognosis [34]. ctDNA seems to present higher sensitivity compared to the plasma protein biomarkers (such as AFP, CEA and PSA), circulating tumor cells and miRNAs [29,35,36]. In hepatoblastomas, ctDNA was considered a more accurate indicator of disease severity and a better biomarker to track the dynamic tumor response compared to AFP [37].

In this study, we profiled CNAs in the tumor tissue from pediatric cancer, including embryonic tumors, followed by further evaluation of CNAs in 1q, *MYCN* and 17p in the circulating cell-free DNA (cfDNA) from plasma samples for long-term disease monitoring through liquid biopsy.

## 2. Materials and Methods

### 2.1. Patient Eligibility and Sample Collection

This study only included Boldrini Children’s Hospital patients who were diagnosed with cancer and who had (patient and/or parents) signed informed consent at the time of the enrolment. Pediatric patients with a pathology-confirmed diagnosis of Wilms tumor (n = 14), neuroblastoma (n = 10), Ewing sarcoma (n = 4), rhabdomyosarcoma (n = 4), leiomyosarcoma (n = 1), osteosarcoma (n = 1) or benign teratoma (n = 1) were included if a plasma sample was available for collection prior to initiation of any curative intervention. Clinical, image-based and pathological data were retrieved from medical records, such as histological diagnosis, stage, metastatic status at diagnosis and follow-up. We used tumor volumes obtained using computed tomography, which derived the measures using the Multislice technique, before and after intravenous injection of iodine contrast, with multiplanar reforms.

For all 35 patients, DNA from the fresh-frozen tumor tissue and ctDNA from the peripheral blood were analyzed before initiation of any treatment. On therapy, or after completion of treatment, blood samples were also collected for 7 patients (2 Wilms tumor, 3 neuroblastoma, 1 rhabdomyosarcoma and 1 osteosarcoma); these time points coincided with surveillance scans over therapy courses.

Tissue samples were then snap-frozen in RNA (Thermo Fisher Scientific, Waltham, MA, USA), and blood samples were collected in an EDTA tube that was processed within 6 h. Samples were centrifuged at 2000× *g* for 15 min at room temperature (RT) for plasma isolation, and then an additional high-speed centrifugation step (3000× *g* for 15 min at RT) was performed to ensure that the plasma was cell-free.

### 2.2. Characterization of the Copy Number Alterations (CNAs) in the Tumors

The GenElute Mammalian Genomic DNA Miniprep Kit (Sigma, St Louis, MO, USA; cat. ID: G1N70-1KT) was used for DNA extraction. DNA samples from tumor tissues were bisulfite converted using an EZ DNA Methylation Kit (Zymo Research, Irvine, CA, USA; cat. ID: D5002) and hybridized in EPIC Beadchip Methylation arrays (Illumina, San Diego, CA, USA). Copy number profiles were retrieved by applying the conumee package [38], which uses the sum of methylated and unmethylated signal intensities compared against healthy reference samples [39]. We determined the can status (diploid, gain or loss) by visual inspection.

For 8 samplecanCNA data were retrieved from the SALSA MLPA (Multiplex Ligation-dependent Probe Amplification; MRC Holland, Amsterdam, The Netherlands) kit using the Probemix P380-B1, following the manufacturer’s instructions. The results were analyzed on Coffalyser.Net.

### 2.3. ctDNA Analysis

Cell-free DNA was extracted from 1 mL of plasma using the QIAamp MinElute ccfDNA Mini Kit (Qiagen, Hilden, Germany; cat. ID: 55204). Cell-free DNA was quantified using the Qubit dsDNA High Sensitive Assay Kit (Invitrogen, Waltham, MA, USA; cat. ID: Q32854).

The dPCR assay was performed using the QIAcuity Probe PCR Kit (Qiagen, Hilden, Germany; cat. ID: 250102) in the QIAcuity One (Qiagen, Hilden, Germany). Reactions were set up for Nanoplate 26K 24-well plates (Qiagen, Hilden, Germany) in a volume of 40 µL consisting of 1× Probe PCR Master Mix (Qiagen, Hilden, Germany), 1× primer–probe mix (Thermofisher, Waltham, MA, USA; cat. ID: Hs05712931, Hs00824796, Hs05506931) as well as 1 ng DNA template. Probes located at *CRABP2* (1q23, to represent 1q), *MYCN* (2p24) and *TP53* (17p13, to represent 17p) were labeled with FAM, and the internal control Copy Number Reference Assay RNaseP (Applied Biosystems, Waltham, MA, USA; cat. ID: 4403328) was labeled with VIC. NTCs contained purified water instead of cfDNA. RNAseP was used for normalization considering unknown and known technical biases, such as pipetting and presence of inhibitors. Our assays provided specific duplexed detection of the target and RNAseP.

Analysis was carried out in the QIAcuity Software Suite, version 2.1.7. 182 (Qiagen, Hilden, Germany), which defined fluorescence thresholds automatically. To enhance the accuracy of concentration measurements, the volume precision factor (VPF), which adjusts for tiny variations in nanoplate geometry, was applied, as recommended by the manufacturer.

### 2.4. Data Analysis

ctDNA was measured in ng/µL per 1 mL of plasma. Values were plotted in violin and correlations graphs using GraphPad Prism, version 9.0 (GraphPad Software). Statistical tests were applied according to the samples’ grouping and distribution. Differences in cfDNA or ctDNA between groups were calculated using the Mann–Whitney test (2 groups) or the Kruskal–Wallis test (3 or more groups). Spearman’s one-tailed test was used to verify whether ctDNA was correlated with tumor characteristics.

## 3. Results

### 3.1. Characterization of 1q, MYCN and 17p Copy Number Status in the Tumors

**Figure 1:**
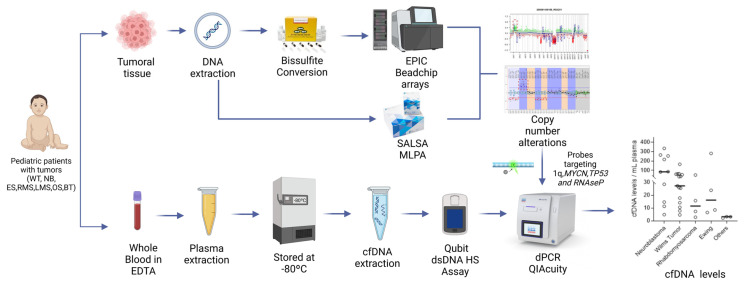
Overview of the study. Tumor tissue and whole blood were collected from pediatric patients diagnosed with cancer. Tumor tissue DNA was extracted and underwent either bisulfite conversion followed by hybridization in the EPIC BeadChip Methylation Arrays or SALSA MLPA assay. CNAs were obtained from either EPIC or MLPA data. Tumors with 1q gain, MYCN gain or TP53 gain/loss were selected for dPCR. Plasma was used for cfDNA extraction and for CNAs’ evaluation. WT, Wilms tumor; NB, neuroblastoma; ES, Ewing sarcoma; RMS, rhabdomyosarcoma; LMS, leiomyosarcoma; OS, osteosarcoma; and BT, benign teratoma.

Patients with a histological diagnosis of a tumor that had primary tumor tissue characterized for CNAs were evaluated using dPCR in the peripheral blood (Figure 1). Characteristics of the 35 patients and tumors were retrieved from medical records. Most patients were diagnosed with neuroblastoma, Wilms tumor, Ewing sarcoma or rhabdomyosarcoma. There was one case of each of leiomyosarcoma, osteosarcoma and benign teratoma (Table 1).

The tumor samples were analyzed for CNAs of 1q, MYCN and TP53. Other CNAs present in these samples were not used for this study. Copy number profiling was derived from EPIC Beadchip arrays that showed 10 out of 26 tumors with a CNA. MLPA reported that four out of eight tumors also had at least one CNA. There were seven cases with 1q gain, nine with MYCN gain, one with TP53 loss and four with TP53 gain.

### 3.2. cfDNA Levels Reflect the Tumor Burden of Pediatric Patients at Diagnosis

**Figure 2:**
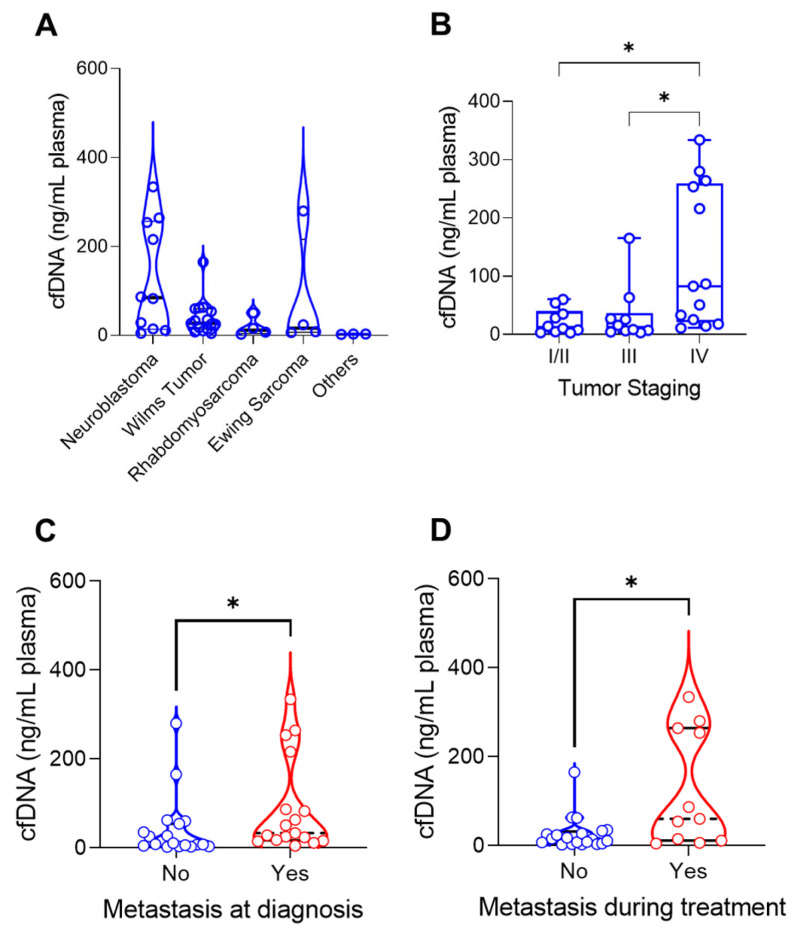
cfDNA levels at diagnosis were evaluated regarding the tumor characteristics. Statistically significant comparison where indicated by “*”. (**A**): cfDNA ng per mL plasma across tumor histologies (*p* = 0.0201, Kruskal–Wallis test). (**B**): cfDNA ng per mL plasma correlated with tumor stage (I/II–IV: *p* = 0.0227, III–IV: *p* = 0.0303). (**C**): Metastatic patients (n = 17) had higher levels of cfDNA at diagnosis compared with nonmetastatic patients (n = 18, *p* = 0.0153, Mann–Whitney U test). (**D**): Patients who developed metastasis during treatment (n = 11) also had higher levels of cfDNA compared to those who did not (n = 19, *p* = 0.0264, Mann–Whitney U test).

cfDNA was extracted from the peripheral blood of patients before they began treatment. The cfDNA levels ranged from 2.16 ng to 334 ng per 1 mL of plasma (Table 1). Comparing the cfDNA levels/mL plasma between tumor types, neuroblastoma had higher median values, followed by Wilms tumor, Ewing sarcoma and rhabdomyosarcoma (*p* = 0.0185) (Figure 2A).

**Figure 3:**
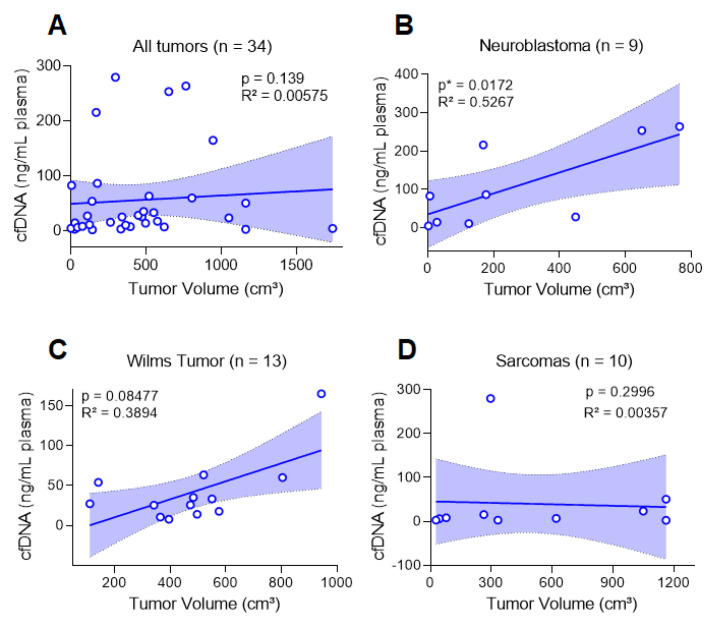
Correlation between cfDNA ng per mL plasma and tumor volume (Spearman’s correlation and linear regression). (**A**): cfDNA ng per mL plasma did not correlate with volume when all tumors were used. Considering each tumor type, (**B**): there was a correlation for neuroblastoma but not for (**C**): Wilms tumor or (**D**): sarcomas (Ewing sarcoma, embryonal rhabdomyosarcoma, osteosarcoma and leiomyosarcoma).

To investigate whether cfDNA had a relation to tumor burden, for all tumors, we compared the tumor characteristics with cfDNA levels. We observed that cfDNA levels were higher in patients with stage IV tumors compared to stages I/II (*p* = 0.0227) and stage III (*p* = 0.0303) in patients with metastasis at diagnosis (*p* = 0.0153) and in those who developed metastasis during treatment (*p* = 0.0264, Figure 2B–D). Although we did not identify an association with tumor histology, for neuroblastoma, we verified a correlation between cfDNA (ng/mL plasma) and the tumor volume (*p* = 0.0172; R^2^ = 0.5267) (Figure 3). For the remaining cases, there was no association between cfDNA levels and clinical characteristics of each tumor type (Table 2).

### 3.3. CtDNA Plasma from Patients at Diagnosis Reflects CNA Status of the Tumor Tissues

**Figure 4:**
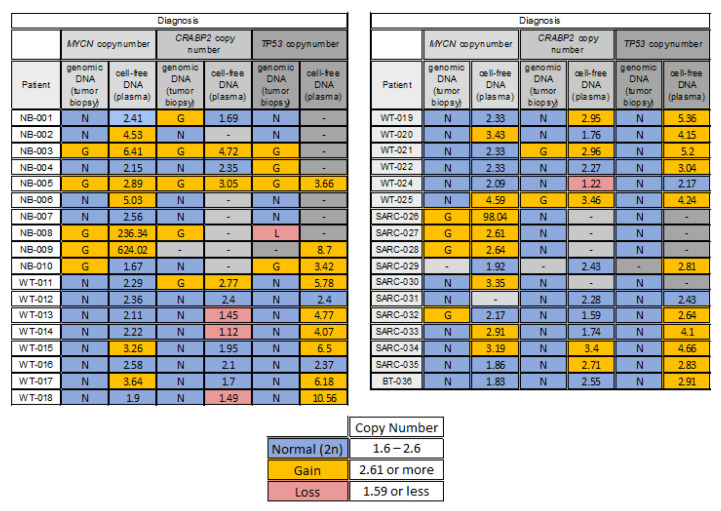
MYCN, CRABP2 (1q) and TP53 (17p) CN for tumor and peripheral blood. NB, neuroblastoma; WT, Wilms tumor; SARC, sarcomas; and BT, benign teratoma. Numbers refer to copy number of the target regions relative to RNAseP. The parameters for gain and loss are also displayed.

At diagnosis, at least one tumor variant was detected in 31 out of 35 (89%) patients. From 79 tumor/cfDNA paired analyses, discordant genomic profiles represented 44%, of which 91% of CNAs were present only in cfDNA and 8.6% only in tumors (Figure 4).

Considering only the ctDNA, the most common alteration at diagnosis was in 17p (83.3%, gain), followed by 1q (48%, of these 66.7% won and 33.3% lost) and MYCN (47%, gain). The MYCN gain was found in six out of ten neuroblastomas analyzed, four out of fourteen in Wilms tumors analyzed and six out of nine sarcomas analyzed.

**Figure 5:**
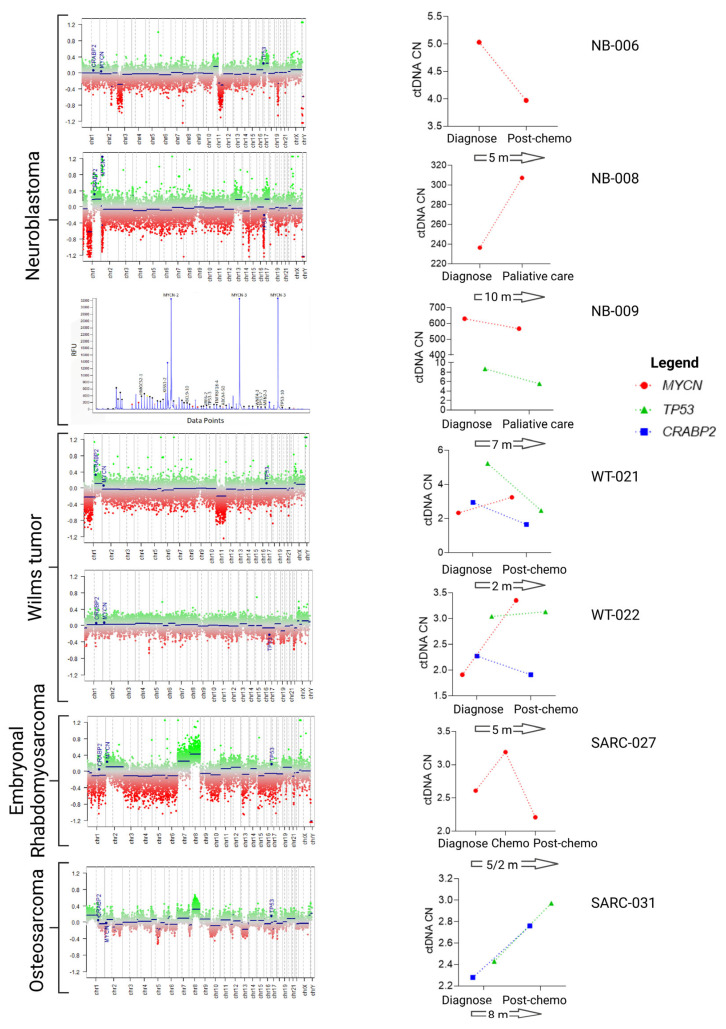
CtDNA CNA in follow-up samples from seven cases. CNAs in the tumor samples at diagnosis were characterized by methylome or MLPA. Genes evaluated in the ctDNA are labeled in blue. The ctDNA levels at diagnosis and in follow-up samples (graphs on the right) were determined by dPCR. The numbers contained within the arrows represent the time (in months) between the analyses performed at diagnosis and at follow-up. Chemo, chemotherapy.

In seven cases, serial plasma samples (one or two) were available for liquid biopsy and we checked for the presence of the CNAs characterized at the time of diagnosis (Figure 5).

Patient NB-006 had a neuroblastoma in which MYCN was not amplified in the sample assayed for CNA. However, the ctDNA showed an increase in the MYCN levels at diagnosis that decreased after 5 months, when ctDNA was re-evaluated. At this point in time, the tumor histology showed that the tumor comprised up to 8% immature neuroblasts, and the remaining cells were considered ganglioneuroma. Similarly, patient SARC-0027 was diagnosed with embryonal rhabdomyosarcoma with MYCN gain at diagnosis. Following chemotherapy, ctDNA levels increased and then decreased at the end of treatment, in agreement with the non-detection of tumor cells by standard tests. Patients NB-008 and NB-009 were both diagnosed with neuroblastoma with high amplification of MYCN. The ctDNA levels remained high or did not change over a period of two evaluations, with both patients eventually dying of the disease.

The three other patients, WT-0021 (Wilms tumor), WT-0022 (Wilms tumor) and SARC-0031 (osteosarcoma), presented heterogeneous ctDNA levels in relation to the tumor tissue regarding the CNAs. WT-0021 had CRABP2 and TP53 gain at diagnosis that were normal in follow-up samples, but MYCN increased during follow-up. The patient is now out of treatment and does not have a detectable tumor. Both WT-0022 and SARC-0031 are considered in remission, although a small increase in the ctDNA has been observed. As these changes are small, they could be related to the limitations of the methodology; this will only be overcome through the analysis of a higher number of samples and by adjusting the technical parameters.

## 4. Discussion

Cancer cells often harbor genomic alterations that include partial or entire gains and losses of chromosomes, large intra-chromosomal inversions or translocations between different chromosomes, or more complex rearrangements. CNAs have been profiled in thousands of tumor samples in virtually all cancer types [9]. In pediatric tumors, among others, recurrent gains involve 1q25.2 (near *CRABP2*), 1q43, *MYCN*, *GLI2*, *MYC*, *TERT*, *BRAF* and 17p11.2, and recurrent losses involve 1p36.13, 4q34.3, *CDKN2A/B*, *PTEN*, 16q24.1, *TP53*, *IGLL5* and *SMARCB1* [5]. In this cohort, we verified that 89% of patients (31 out of 35) had at least one CNA identified in the tumor.

Liquid biopsy technologies have emerged as a minimally invasive approach useful when tumor tissue is inadequate or non-existent. Components derived from the tumor, such as the cell-free nucleic acids, might be measured as cfDNA or ctDNA, providing a potential tumor biomarker [40]. Patients with cancer usually have high concentrations of cfDNA in the peripheral blood [41], which may be dependent on tumor type. In our cohort, the median cfDNA ng/mL of plasma was highest for neuroblastoma, followed by Wilms tumor, Ewing sarcoma and rhabdomyosarcoma. In a cohort of 45 pediatric patients, cfDNA levels were also higher in neuroblastoma compared with osteosarcoma, rhabdomyosarcoma and Wilms tumor [42], which could be related to tumor biology. Moreover, cfDNA levels correlated with neuroblastoma tumor volume, but not for Wilms tumor or sarcomas. However, a greater number of cases need to be evaluated before drawing a more definitive conclusion. Neuroblastomas are aggressive tumors. In particular, those with *MYCN* amplification present a poor survival rate, even for localized disease [43]. The higher cfDNA levels in neuroblastoma could also reflect the high disease burden present in patients with metastatic disease [42]. Accordingly, independent of tumor type, we found that cfDNA levels were associated with metastasis at diagnosis and metastasis during treatment, with the ctDNA levels increasing strongly in the presence of distant metastases as well as with an advance of tumor stage.

To determine the source of ctDNA, which is a fraction of cfDNA, is a major limitation for the implementation of dPCR as a routine test for pediatric cancer. Pediatric solid tumors are known to show higher numbers of structural variants compared to point mutations [5]. the recurrent mutated genes are usually long, with the transcript divided into several exons, but no hotspots are reported in pediatric tumors, such as in the case of some tumor suppressor genes: *RB1* (27 exons), *WT1* (12 exons), *TP53* (11 exons), *PTEN* (10 exons). Some exceptions include mutation in exon 3 from *CNNTB1* in hepatoblastoma [44,45] and Wilms tumors [46] and *H3K27* in central nervous system tumors [47]; these are good candidates for liquid biopsy and are being tested in several tumors [48]. Due to the small quantity of recovered cfDNA and the characteristics of the mutational profile in pediatric tumors, the application of a target methodology, such as dPCR, can only be of limited use. Nevertheless, the use of CNAs in the peripheral blood can point to the presence of ctDNA [49]. ctDNA are fragments of around 166 bp DNA released in the blood by the tumor cells and they contain the original tumor molecular alterations. Digital PCR is a highly sensitive and linear method to assess the copy number in tumor-derived cfDNA from plasma. The methodology detects the absolute concentration of targeted sequences and a diploid reference gene [18,27]. In this paper, we established the identification of CNAs in *CRABP2* and *TP53* as surrogates for 1q and 17p, respectively, as well as that of *MYCN* by dPCR, aiming toward the implementation of a routine copy number status assessment.

Considering that we used probes located at TP53 as a surrogate for 17p, the finding that most samples presented 17p gain should be taken with caution as this high CNA number has not been found in the literature. The probe from this assay was located at the first intron from TP53. Non-coding regions are less resistant than coding regions to somatic alterations, which could explain, at least in part, the high number of alterations.

From 79 tumor/cfDNA paired analyses, discordant genomic profiles represented 44%, in which 91.4% of CNAs were present only in cfDNA and 8.6% only in tumors. Tumor heterogeneity might also have accounted for the observed differences. Single tumor biopsies do not always reflect clonal heterogeneity within the primary tumor, and spatial and temporal heterogeneity have been described for several cancer entities, including Wilms tumors [50] and neuroblastoma [51,52]. Furthermore, dPCR presents limitations, along with the divergence between tumor tissue and ctDNA, as reported here and for *MYCN* status in patients with neuroblastoma [53], 1q status and Wilms tumors [42,54] and others. These limitations must be carefully evaluated before the implementation of this methodology in a routine setting.

We found *MYCN* gain in the peripheral blood in 16 cases (46%); this is similar to other studies that reported 42% of 52 patients with neuroblastoma having *MYCN* gain [53,55].

Although we found some CNAs in Ewing sarcoma, the assessment of the driver translocation, which involves the family EWS, constitutes a more attractive biomarker to be pursued. In the literature, up to 91% of 102 cases had detectable ctDNA before the start of chemotherapy [42,56].

For seven patients, we observed changes in ctDNA levels during follow-up. Monitoring these changes in patients undergoing treatment could lead us to define a response-based risk stratification. The feasibility of such a proposal was tested in a wide range of pediatric solid tumors, and the researchers proposed a prospective study within each pediatric tumor to validate the use of ctDNA in prognostics [42].

This pilot study in pediatric tumors demonstrated that both cfDNA and CNAs can represent feasible biomarkers for a variety of solid tumor types and clinical indications. We demonstrated an association between tumor stage, metastasis at diagnosis, metastasis during treatment and tumor volume regarding neuroblastoma, in a routine setting. The differences between tumor genomic profile and cfDNA profile are obstacles that could be caused by subclonal events or technical limitations. In accordance with the current literature, we believe that CNAs may become relevant biomarkers to be explored for liquid biopsy to monitor tumors where these CNAs are relevant. However, the proven clinical utility of CNAs as biomarkers for liquid biopsy, as well as the benefits of knowledge of ctDNA levels, can only be ascertained through larger studies of ctDNA in pediatric patients with cancer. We believe that this study lays the foundation for such future studies.

## Figures and Tables

**Table 1:** Characteristics of the patients and tumors.

**Clinical Variable**	**Patients, No. (%)**
**Patients**	35 (100%)
**Sex**	
Male	18 (51%)
Female	17 (49%)
**Age, years**	
1–10	29 (83%)
>10	6 (17%)
**Diagnosis**	
Wilms tumor	14 (40%)
Neuroblastoma	10 (29%)
Ewing Sarcoma	4 (11%)
Rhabdomyosarcoma	4 (11%)
Others	3 (9%)
**Tumor Volume**	
<50	5 (14%)
50–100	1 (3%)
101–500	16 (46%)
501–1000	8 (23%)
>1000	4 (11%)
Unknow	1 (3%)
**Clinical Stage**	
I	1 (3%)
II	9 (26%)
III	10 (29%)
IV	13 (37%)
Unknow	2 (6%)
**Metastasis at diagnosis**	
Yes	17 (49%)
No	18 (51%)
**Metastasis during the treatment**	
Yes	11 (31%)
No	19 (54%)
Unknow	5 (14%)
**cfDNA levels (ng/mL plasma)**	
<10	10 (29%)
10–50	13 (37%)
51–100	6 (17%)
>100	6 (17%)

**Table 2:** Characteristics of the tumors.

**Clinical Variable**	**Patients, No. (%)**			**Statistical Test**
**Clinical Stage**	WT	NB	Sarcomas	
I	0	0	1 (10%)	Not applied
II	6 (43%)	2 (20%)	1 (10%)	
III	5 (36%)	0	5 (50%)	
IV	3 (21%)	8 (80%)	2 (20%)	
Unknow	0	0	1 (10%)	
**Correlation cfDNA levels vs. tumor staging**				
I/II vs. III	*p* = 0.99	-	*p* = 0.66	Mann-Whitney test and Kruskal-Wallis test.
I/II vs. IV	*p* = 0.99	*p* = 0.18	*p* = 0.05	
III vs. IV	*p* = 0.99	-	*p* = 0.32	
**Correlation cfDNA levels vs. tumor volume**				
	*p* = 0.08	*p* = 0.02	*p* = 0.29	Spearmen test.
**cfDNA levels and metastasis vs. no-metastasis at diagnosis**				
	*p* = 0.70	#	*p* = 0.12	Mann-Whitney test. # Statistics cannot be performed.
**cfDNA levels and metastasis vs. no-metastasis during treatment**				
	*p* = 0.94	#	*p* = 0.50	Mann-Whitney test. # Statistics cannot be performed.

## Data Availability

Not applicable.
