# Correction: Ruas et al. Somatic Copy Number Alteration in Circulating Tumor DNA for Monitoring of Pediatric Patients with Cancer. *Biomedicines* 2023, *11*, 1082

**DOI:** 10.3390/biomedicines13122872

**Published:** 2025-11-25

**Authors:** Juliana Silveira Ruas, Felipe Luz Torres Silva, Mayara Ferreira Euzébio, Tássia Oliveira Biazon, Camila Maia Martin Daiggi, Daniel Nava, Mayra Troiani Franco, Izilda Aparecida Cardinalli, Alejandro Enzo Cassone, Luiz Henrique Pereira, Ana Luiza Seidinger, Mariana Maschietto, Patricia Yoshioka Jotta

**Affiliations:** 1 Research Center, Boldrini Children’s Hospital, Campinas 13083-884, SP, Brazil; ruasjulianas@gmail.com (J.S.R.); felipeluztorres@outlook.com (F.L.T.S.); ma.euzebio@gmail.com (M.F.E.); tassiabiazon@gmail.com (T.O.B.); analuseidinger@gmail.com (A.L.S.); marianamasc@gmail.com (M.M.); 2 Genetics and Molecular Biology, Institute of Biology, State University of Campinas, Campinas 13083-862, SP, Brazil; 3 Boldrini Children’s Hospital, Campinas 13083-210, SP, Brazil; camilammdaiggi@gmail.com (C.M.M.D.); danielnava@gmail.com (D.N.); mayra.franco@boldrini.org.br (M.T.F.); izilda.cardinalli@boldrini.org.br (I.A.C.); alejandro.cassone@boldrini.org.br (A.E.C.); drluizhpereira@gmail.com (L.H.P.)

## Text Correction

There was an error in the original publication [1]. The textual description of Figure 4 in the Results section, in the Subsection “*CtDNA Plasma from Patients at Diagnosis Reflects CNA Status of the Tumor Tissues*”, paragraph 2, mistakenly reported the MYCN gain as “six out of nine neuroblastomas” and “six out of eight sarcomas analyzed” instead of “six out of ten neuroblastomas” and “six out of nine sarcomas analyzed”, respectively.

The corrected paragraph is as follows:

Considering only the ctDNA, the most common alteration at diagnosis was in 17p (83.3%, gain), followed by 1q (48%, of these 66.7% won and 33.3% lost) and MYCN (47%, gain). The MYCN gain was found in six out of ten neuroblastomas analyzed, four out of fourteen in Wilms tumors analyzed and six out of nine sarcomas analyzed.

The authors state that the scientific conclusions are unaffected. This correction was approved by the Academic Editor. The original publication has also been updated.
